# It’s five o’clock somewhere: An examination of the association between happy hour drinking and negative consequences

**DOI:** 10.1186/1747-597X-9-17

**Published:** 2014-04-23

**Authors:** Julie Marie Baldwin, John M Stogner, Bryan Lee Miller

**Affiliations:** 1 Department of Criminal Justice, University of Arkansas at Little Rock, 2801 S. University Avenue, Ross Hall 5th Floor, Little Rock, AR 72204-1099, USA; 2 Department of Criminal Justice & Criminology, University of North Carolina at Charlotte, 5062 Colvard North, Charlotte, NC 28223, USA; 3 Department of Criminal Justice and Criminology, Georgia Southern University, P.O. Box 8105, Statesboro, GA 30460, USA

**Keywords:** Happy hour, Alcohol price, Increased alcohol consumption, Negative consequences, Alcohol policy

## Abstract

**Background:**

This study aims to understand which young adults’ drinking behaviors change in the presence of happy hour specials, the ways in which they change, and whether a link exists between happy hour drinking behavior and negative outcomes.

**Methods:**

Using data collected from bar-going respondents (n = 1,423) within a print survey administered to a general college sample (n = 2,349), we identify significant differences in changes in happy hour behavior between demographic groups using χ^2^ tests and determine whether this behavior is related to six negative alcohol-related outcomes using logistical and ordinary least squares regression models with a variety of controls, including age of onset and frequency of use.

**Results:**

Women, students under 21, non-athletes, members of Greek-affiliated organizations, more affluent and unemployed students, and students living on campus were more likely to change their drinking behavior in the presence of happy hour specials. In general, the most robust predictors of negative events are gender, alcohol use frequency, age of alcohol use onset, and increasing drinking due to happy hours/bar specials. While it was linked to various negative and illegal *behaviors*, altered happy hour drinking was not associated with an increased likelihood of an alcohol-related *arrest*.

**Conclusions:**

This study lends support to the idea that alcohol price specials should be regulated in an effort to reduce high consumption and alcohol-related negative consequences. Future research into the relationship between happy hour drinking and negative outcomes is necessary and should examine the impact of happy hour advertisements, different types of specials, and the timing of happy hours.

## Introduction

Research has been consistent in finding a relationship between “binge drinking” (broadly defined as consuming an excess of alcoholic beverages in a single episode) and numerous negative consequences. Specifically, sexual assault victimization, violent behavior, property damage, risky sexual behavior, poor academic performance, contact with law enforcement, physical injury, and death have been linked to binge drinking behaviors in young adult populations e.g., [1-5]. Binge drinking can also negatively affect those not engaging in the behavior, as they may have to care for an inebriated peer, have their own activities interrupted, or be the victim of a traffic accident or sexual assault facilitated by another’s intoxication [3,4].

Since the late 1970s, researchers in a variety of fields have examined the relationship between alcohol prices and alcohol consumption by youth and young adults. Findings from econometric studies are somewhat mixed [6] but generally indicate that alcohol consumption follows the downward sloping demand curve law where as a product price increases, the quantity demanded decreases [6,7]. Results in public health studies have been fairly consistent in finding the inverse relationship where consumption increases as the general price of alcohol decreases [7,8]. Overall, alcohol price seems to affect the drinking behavior of the young adult population more than other age groups [8,9], but it also significantly affects adolescent drinking [10].

The price of alcohol may be reduced due to market competition between manufacturers, competition between retailors, decreases in liquor and/or beer tax, and, most relevant to the current study, bars and restaurants utilizing happy hour specials to increase patronage during traditionally non-peak hours. Happy hour is a term for a set period of time, often advertised, when an establishment serves alcohol at a discounted rate. Several studies have focused on college populations and happy hours/drink specials, which is warranted because national studies in the U.S. have reported that more than 40% of college students engaged in binge drinking in the last year [11-14]. Further, approximately 1,700 deaths and an additional 600,000 traumas among U.S. college students result from alcohol use each year [2].

Research has primarily found an increase in alcohol consumption and drinking intentions of college students during happy hour specials with some studies noting differences by sex and age. In the U.S., Kuo *et al*. (2003) found that low alcohol prices and frequent alcohol promotions by on- and off-campus establishments were related to higher self-reports of binge drinking by college students [9]. Thombs *et al*.’s (2008) field study in the U.S. also found the inverse relationship between alcohol specials and consumption [15]. Their natural observation of patrons exiting bars found that those who bought alcohol on special were 4.38 times more likely to have a BAC of at least 80 mg/dl than those who did not. While males’ consumption was higher than females, the levels of intoxication achieved were similar across sexes. Individuals below the legal drinking age (under 21) had higher levels of intoxication than patrons of the legal drinking age [15]. Other studies in the U.S. show a positive correlation between students’ perceptions of their drinking intentions and behavior and alcohol promotions and advertising [3,16].

Although concerns exist about generalizing results of college students in their natural drinking environments, studies of other populations and in simulated environments echo previous findings. For example, an Australian study of community football clubs found that happy hour promotions were associated with club members being more than twice as likely to consume alcohol to excess [17]. Additionally, Babor *et al*.’s (1978) experiment found that both casual and heavy drinkers consumed more than twice as much alcohol in simulated happy hours in comparison to consumption in the absence of alcohol specials [18].

Because of the links between increased alcohol consumption and negative consequences and between the decreased cost of alcohol and increased consumption, some researchers e.g., [7] have argued that policymakers should increase alcohol prices in an effort to decrease consumption and reduce the negative consequences associated with high consumption. Studies have found that increasing beer excise taxes in the U.S. was one of the most effective mechanisms in reducing drinking and driving for youths [19-21]. In their review of the literature, Chaloupka *et al*. [7] concluded that an increase in alcohol prices and the financial costs of penalties (e.g., increases in the actual purchase price of alcohol, increases in the expected legal costs of driving under the influence from more severe laws), as well as further limiting availability, reduces drinking and driving and its consequences in the entire population [7]. Beer tax has a strong inverse relationship with workplace injuries [22], and increases in liquor and beer tax have also been linked to decreases in gonorrhea and syphilis rates [23]. However, the effect of increased alcohol prices on cirrhosis mortality is mixed [24-26].

Research in this area has the potential for important policy implications. Several countries, states, and localities have attempted to ban or regulate happy hour promotions including how and whether they are advertised. For example, the Australian Code of Practice for Responsible Promotion of Liquor Products restricts advertisements for happy hours, although research reveals that many venues regularly breach both the spirit and the letter of the code [27]. In the U.S., legislation has varied on alcohol prices and the advertisement of alcohol promotions. Currently, Utah has banned happy hours [28], and Massachusetts has banned bars from offering drinks specials [29], resulting in bar owners offering specials on food instead of alcohol. Oregon allows happy hour specials but prohibits the advertising of happy hours and prohibits advertising of purchasing more than one drink at a special price (e.g., buy one get one free, two for one) [30]. Virginia also permits happy hour specials, but establishments cannot advertise specific drink specials, have happy hours between certain hours, and have limits on the number of drinks an individual can possess at one time [31].

Some states within the U.S. have recently amended their alcohol price and promotion legislation. Although Virginia still has restrictions in place (previously presented), it repealed a previous ban in January of 2014 that prohibited advertising any happy hour specials through electronic and print media (e.g., Internet, radio, TV, newspaper, social media) [31]. In 2012, Kansas repealed its 26-year-old happy hour ban [32]. Pennsylvania recently increased the timeframe in which happy hour specials can be offered but limits the number of hours per week, restricts the timeframes, and limits the number of drinks an individual can have at any point in time [33].

Although research has individually explored the relationship between alcohol pricing and consumption or intoxication levels, as well as the relationship between consumption and negative consequences, research has yet to examine the relationship between happy hour drinking behavior and negative outcomes. The majority of extant studies focus on overall prices or daylong drink specials without considering time-limited reductions in costs that may drive both increases in total alcohol consumption and increases in the speed of consumption. Short windows of lower-priced alcohol may lead financially constrained consumers to drink a larger quantity of alcohol in a short period of time to maximize the perceived return on their spending. As such, this study aims to understand whose drinking behaviors change in the presence of happy hour specials, the way in which they change, and, further, whether there is a link between happy hour drinking behavior and negative outcomes such as intoxicated driving, alcohol-related arrests, risky sexual behavior, fighting, and general alcohol-related problems.

## Methods

### Data

The overall data collection effort, titled "An Examination of Undergraduate Substance Use and High-Risk Behavior," was reviewed for ethical concerns by the Institutional Review Board of Georgia Southern University, United States. After minor modifications, it received approval (IRB protocol H12032) during the fall of 2011. In January through March of 2012, a print survey was administered to 2,349 students attending classes at the University. The university is situated in a small town with numerous establishments that serve alcohol within a short walking distance of campus. Given the large university population relative to the size of the town’s non-collegiate population (roughly 2:1), most of these establishments primarily serve the college student population. However, the university is located within a “dry county”, meaning liquor drinks can be bought at a bar and consumed on premise, but liquor bottles cannot be purchased at a store within the county and taken home to consume.

In order to obtain the sample, 40 classes were randomly selected from two strata: 15 high enrollment courses (100 or more students) and 25 moderate enrollment classes (30 to 99 students). Due to the feasibility issues that exist in administering a paper survey in non-traditional classrooms, laboratory, online, and kinesiology courses were removed from the sampling frame. A single research assistant administered the survey in each selected class on a date chosen by the instructor. In cases where instructors denied access to their classes, randomly selected classes from their respective strata replaced these courses. Students were not required to participate in the study nor were any attempts made to contact absent students. The resulting sample of 2,349 students represents more than 80% of those enrolled in the courses at the end of the first week of classes.^a^

The full sample was largely representative of the student population. Whereas the student population was 51.5% female, 65.5% White, 25.0% African American, and 3.8% Hispanic, the sample was 51.6% female, 68.9% White, 24.4% African American, and 2.8% Hispanic with a small portion of students falling into other race/ethnicity categories. The median family income category of the full sample was $75,000 to $99,999. Respondents reported a mean age of 20.06, and 2.8% self-identified as gay, bisexual, or transgender. Members of Greek-affiliated organizations constituted 15.7% of the sample, and 5.3% were student athletes. The sample had a mean GPA of 3.017; however, 339 were first semester students and have not yet established a GPA.

As the interest of the present study lies solely in the impact of happy hour drinking, we only focus on those that reported having had the opportunity to engage in the behavior in the past. The analytic sample was restricted to those that either were old enough to legally drink, had fake identification, or self-reported other access to alcohol in a bar or restaurant. This eliminated approximately 36.7% of the initial sample. The sample was further restricted to alcohol users. This restriction removed 4.1% of the remaining sample, resulting in a total sample size of 1,423 respondents (60.6% of the original sample). Compared to those eliminated from the full sample, the analytic sample contained a smaller percentage of females (49.4%; χ^2^ = 7.711, 1 df, p = .005), fewer African Americans (16.6%), a larger percentage of Whites (77.3%; χ^2^ = 127.889, 2 df, p < .001), and more students in Greek-affiliated organizations (21.4%; χ^2^ = 86.806, 1 df, p,.001). The mean age for the analytic sample is slightly higher (20.60 years old; t = 11.022, 2339 df, p < .001); however, it is still less than the legal drinking age. We attribute this to the 788 students that reported having used false identification at a bar or club. The GPA of the analytic sample was slightly higher (3.060) than those excluded (2.992). This difference was significant (t = 2.429, 2008 df, p = .015) but small in magnitude. All other demographic characteristics are not significantly different from those of the full sample.

### Measures

#### Happy hour drinking

A single item asked respondents “Compared to normal drinking, how does your drinking change when you attend a bar with a happy hour or drink special?” Respondents selected one of the following: “I drink more” (32.5%), “I drink more quickly” (8.6%), “I drink more and more quickly” (23.0%), “My drinking doesn’t change” (32.5%), or “I drink less and/or more slowly” (3.4%). For the main analysis, the first three responses were collapsed into a single category representing those who increased drinking during happy hours (64.1%), and the final two were collapsed into a category representing those who do not increase their drinking during happy hours (35.9%). These measures represent any type of *increase* in drinking behaviors and not the *amount* of drinking. This is consistent with our focus on the effect of happy hour specials rather than overall alcohol consumption.

#### Negative outcomes

Six distinct negative outcomes related to alcohol use in the last year were evaluated. Five of these were dichotomous measures that indicated whether a specific alcohol-related event occurred. First, a self-reported driving under the influence measure was created from a question that asked respondents if they had driven a car/truck on the road after five or more drinks. Second, we included a measure that assessed whether the respondents had any alcohol-related arrests. Those having been arrested or cited for driving under the influence, open container violations, using a fake ID to obtain alcohol, underage alcohol use, or public drunkenness were coded as having an alcohol-related arrest. Third, we focused solely on arrest or citation for driving under the influence. Fourth, respondents were asked whether they had engaged in unprotected sexual intercourse with a stranger while intoxicated. Fifth, respondents were asked whether they had engaged in “malicious fighting” while intoxicated with clarification that it did not include “play fighting, wrestling, or sparring.” Finally, a last-year alcohol-related problems scale similar to that previously used by Maney, Higham-Gardill, and Mahoney (2002) [34] was created (α = .814). Ten Likert-type items with six option choices, ranging from “not a problem” (1) to “severe” (6), assessed the severity of problems that resulted from alcohol use. Items asked respondents whether and to what degree alcohol had affected familial relationships, friendships, romantic relationships, physical health, emotional well-being, and finances, as well as created legal problems. This scale is intended for populations such as this one and attempts to determine the degree to which alcohol more generally created problems in a person’s life.

#### Alcohol use frequency

The frequency of alcohol consumption is included as a control in the regression models and is measured with the alcohol item from a section that asked respondents to indicate “the number of days that you have used each of the following substances recreationally ***in the last 30 days”*** (emphasis in survey). Respondents were provided six options on an ordinal scale ranging “from none” (0) to “twenty or more days” (5).

Note, no measure of average drinks consumed in each drinking episode is contained within the data. The age of first alcohol use was quantified as were demographic factors. The use of other substances was not included in the analysis because a cross-sectional survey design prevents us from establishing whether the negative outcomes preceded the use of other drugs. Whereas alcohol use necessarily has to precede driving under the influence or fighting while intoxicated, marijuana use and other drug use may have only occurred following those events; thus, they would be inappropriate controls.

### Analytic strategy

We first present analyses focused on determining which students change their behavior in the presence of happy hours and alcohol specials. We present the proportion of each demographic category that reports happy hour specials increase their drinking and identify significant differences using χ^2^, Mann–Whitney U, and t-tests as appropriate. We then individually regress each of the six negative alcohol-related outcomes onto whether happy hours increase alcohol consumption and a variety of controls including the respondent’s frequency of alcohol use and the age at which they initiated alcohol use. We utilize logistic regression models for the first five outcomes and ordinary least squares regression for the alcohol-related problems scale.

## Results

Table 1 displays the percentages of each demographic group by self-reported response to happy hours or drink specials. Potential differences in the traits listed in the upper portion of the table were assessed using χ^2^ analyses. As can be seen in the first two rows, a significantly larger portion of women reported increases in their drinking than men. No significant differences emerged between racial groups. Significantly more of those not yet old enough to legally drink alcohol reported that happy hours and specials increased their drinking than each of the three age categories above the legal threshold for alcohol consumption.^b^ Altered drinking during happy hours was reported by significantly more freshmen and sophomores than juniors and seniors. Additionally, significantly fewer student athletes altered their drinking than non-athletes, and more individuals in Greek-affiliated organizations changed their behavior in comparison those not in a fraternity or sorority. There was a significant association between grade point average (GPA) and changes in happy hour drinking. More specifically, fewer of those with GPAs between 2.01 and 3.00 altered their drinking due to happy hour than those in other ranges. Although a much smaller portion of the married individuals reported altered drinking than single participants, the difference was not significant.

**Table 1:** Differences in happy hour drinking across demographic categories

**Characteristics**	**N**	**Happy hour increases drinking**	**Happy hour does not increase drinking**	**χ**^**2**^**(df)**	**p value**
**Gender**					
Male	679	59.8% (460)	40.2% (273)	10.878 (1)	.001
Female	674	68.4% (461)	31.6% (213)		
**Race**					
African American	220	65.5% (144)	34.5% (76)	1.643 (2)	.440
White	1030	64.6% (665)	35.4% (365)		
Other	78	57.7% (45)	42.3% (33)		
**Age**					
17-20	793	77.4% (614)	22.6% (179)	149.815 (3)	<.001
21-22	421	47.0% (198)	53.0% (223)		
23-25	85	40.0% (34)	60.0% (51)		
26 or older	53	39.6% (21)	60.4% (32)		
**Class year**					
Freshman	441	77.8% (343)	22.2% (98)	105.603 (3)	<.001
Sophomore	351	72.4% (254)	27.6% (97)		
Junior	297	49.8% (148)	50.2% (149)		
Senior	243	46.5% (113)	53.5% (130)		
**GPA**					
3.01 – 4.0	594	65.8% (391)	34.2% (203)	13.474 (4)	.009
2.01 – 3.0	534	59.9% (320)	40.1% (214)		
1.01 – 2.0	88	61.4% (54)	38.6% (34)		
0.0 – 1.0	5	100% (5)	0.0% (0)		
No GPA	134	73.9% (99)	26.1% (35)		
**Student athlete**					
No	1293	64.9% (454)	35.1% (31)	6.252 (1)	.012
Yes	61	49.2% (839)	50.8% (30)		
**Fraternity/Sorority**					
No	1063	62.7% (667)	37.3% (396)	4.119 (1)	.042
Yes	292	69.2% (202)	30.8% (90)		
**Employed**					
No	900	66.3% (597)	33.7% (303)	6.818 (2)	.033
Part-time	399	60.9% (243)	39.1% (156)		
Full-time	55	52.7% (29)	47.3% (26)		
**Marital status**					
Single	1319	64.5% (851)	35.5% (468)	3.427 (1)	.064
Married	31	48.4% (15)	51.6% (16)		
**Family income**					
Over $100,000	619	65.6% (406)	34.4% (213)	12.013 (4)	.017
$75,000 – $99,999	216	68.5% (148)	31.5% (68)		
$50,000 – $74,999	242	65.7% (159)	34.3% (83)		
$25,000 – $49,999	155	52.9% (82)	47.1% (73)		
Under $24,999	86	59.3% (51)	40.7% (35)		
**Living arrangements**					
With parents/family	77	55.8% (43)	44.2% (34)	61.826 (4)	<.001
Campus dormitories	405	78.8% (319)	21.2% (86)		
Greek housing	25	68.0% (17)	32.0% (8)		
Rented apartment	812	59.1% (480)	40.9% (332)		
Home owner	26	30.8% (8)	69.2% (18)		
				Mann–Whitney U (standardized)	p value
**Alcohol use frequency (Median)**		6-9 days per month	3-5 days per month	4.713	<.001
				t-value (df)	p value
**Age of first alcohol use (M (SD))**		15.79 (2.08)	16.31 (2.61)	4.013 (1324)	<.001

A significantly larger percentage of those from higher income families reported that their behavior was influenced by happy hours and bar specials than those from less affluent families. A different relationship was seen when examining the students’ own employment. Significantly fewer of those working full-time and part-time self-reported changes in behavior compared to those who were unemployed. Living arrangements were strongly associated with happy hour behavior changes. Far more of those living in campus dormitories reported altered drinking than those living in any other setting. Finally, a Mann–Whitney U test indicated that increased happy hour drinking was reported significantly more among those that reported using alcohol more frequently. A t-test revealed that the average age of alcohol initiation was significantly lower among those that reported altered drinking during happy hours.

**Figure 1:**
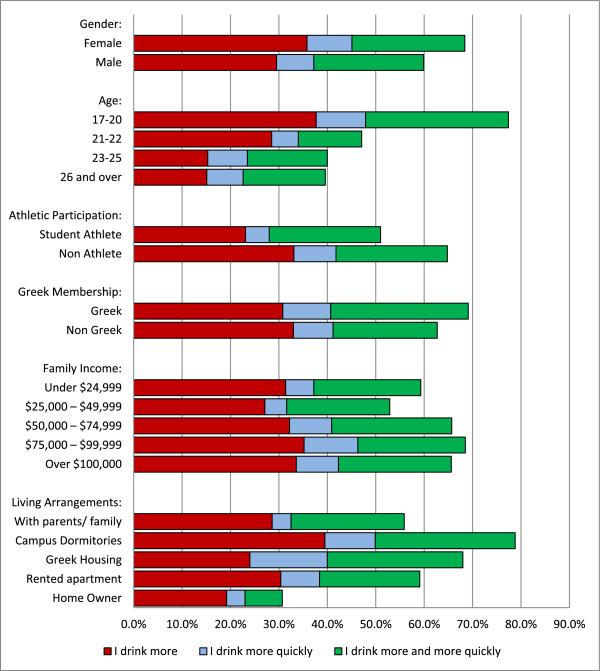
Self-reported changes in drinking behavior during happy hour.

This information is presented graphically in Figure 1. In addition to depicting the portion of each group that altered their drinking during happy hours and bar specials, the figure shows how each group reported it affecting their drinking. Among other things, the figure demonstrates that happy hours most affected the drinking of those that cannot legally drink and those whom live on the university campus. Alternatively, it least affected the behavior of older students and homeowners.

Tables 2 and 3 present the six regression models predicting negative events related to alcohol use. Each model regressed an outcome on twelve factors: gender (coded 0 = female, 1 = male), race (coded 0 = White, 1 = non-White), age, athletic participation, membership within a Greek-affiliated organization, employment status, family income, marital status, living arrangements, frequency of alcohol use, age at which a person first used alcohol, and whether a person increased alcohol consumption during happy hours. In general, the most robust predictors of negative events appeared to be gender, frequency of alcohol use, age of onset, and increasing drinking due to happy hours/bar specials.

**Table 2:** Regression models evaluating happy hour behaviors and illicit behaviors/arrests

	**Logistic regression models**								**Rare events logistic regression model**			
	**Driving under the influence**				**Alcohol-related arrest**				**DUI citation/arrest**			
**Characteristics**	**O.R.**	**95% CI**	**Wald χ**^**2**^	**p**	**O.R.**	**95% CI**	**Wald χ**^**2**^	**p**	**O.R.**	**95% CI**	**Wald χ**^**2**^	**p**
Gender (0 = F,1 = M)	2.44	1.50-3.96	13.07	<.01	1.77	1.29-2.44	12.17	<.01	2.17	.75-6.25	2.04	.16
Race (1 = Non-White)	.67	.36-1.27	1.51	.22	.38	.23-.64	13.68	<.01	.50	.10-2.58	.69	.41
Age	1.06	.97-1.16	1.80	.18	1.09	1.03-1.17	7.42	.01	1.56	.71-3.46	1.21	.28
Student athlete	1.07	.36-3.10	.01	.91	1.28	.63-2.58	.47	.50	2.46	.50-12.13	1.22	.27
Fraternity/Sorority	.80	.45-1.43	.56	.46	1.36	.94-1.97	2.65	.11	1.08	.36-3.22	.02	.90
Employed‡												
Part-time	1.90	1.19-3.05	7.11	.01	1.21	.85-1.72	1.15	.29	1.31	.40-4.01	.20	.66
Full-time	1.33	.41-4.30	.23	.63	.92	.38-2.21	.04	.86	16.12	2.62-99.10	9.00	<.01
Family income	1.04	.93-1.16	.37	.55	1.06	.97-1.14	1.73	.19	1.02	.79-1.32	.02	.88
Married	1.33	.32-5.51	.15	.70	1.31	.44-3.91	.24	.63	.00	.00-19424	.00	1.00
Off-campus housing	1.31	.75-2.31	.90	.35	.90	.62-1.31	.30	.59	.72	.22-2.42	0.28	.60
Alcohol use frequency	1.32	1.12-1.56	11.29	<.01	1.14	1.02-1.28	5.22	.03	1.20	.83-1.73	.97	.33
Age of alcohol onset	.89	.83-.95	12.19	<.01	.91	.86-.96	12.97	<.01	.87	.74-1.03	2.59	.11
Happy hour drinking	1.88	1.12-3.15	5.67	.02	1.24	.88-1.74	1.45	.23	1.03	.34-3.17	.00	.96
Constant	.01				.05				.00			
Model χ^2^	67.01				79.88				66.23			
Pseudo R^2^	.128				.106				.08			

**Table 3:** Regression models evaluating happy hour behaviors and negative consequences

	**Logistic regression models**								**OLS linear regression**			
	**Unprotected sex while drinking**				**Fighting while drinking**				**Alcohol-related problems**			
**Characteristics**	**O.R.**	**95% CI**	**Wald**	**p**	**O.R.**	**95% CI**	**Wald**	**p**	**b**	**se**	**β**	**p**
Gender (0 = F,1 = M)	1.54	1.19-1.99	10.75	<.01	3.65	2.22-5.98	26.34	<.01	.00	.04	.00	.95
Race (1 = Non-White)	.88	.63-1.22	.60	.44	1.06	.61-1.89	.05	.84	-.01	.04	.00	.92
Age	1.01	.95-1.07	.11	.75	1.01	.91-1.11	.01	.93	-.01	.01	-.04	.35
Student athlete	.99	.55-1.81	.00	.99	1.11	.42-2.96	.04	.84	-.02	.08	-.01	.80
Fraternity/Sorority	.98	.71-1.34	.02	.89	1.12	.66-1.87	.17	.68	.06	.04	.04	.19
Employed‡												
Part-time	.99	.74-1.32	.01	.93	1.25	.78-1.99	.86	.36	-.02	.04	-.01	.64
Full-time	1.63	.84-3.19	2.06	.16	.24	.03-1.91	1.81	.18	-.07	.10	-.02	.45
Family income	.95	.89-1.01	2.53	.12	1.02	.92-1.14	.15	.70	.00	.01	.01	.80
Married	1.41	.57-3.49	.54	.47	2.47	.63-9.71	1.81	.19	-.90	.13	.02	.51
Off-campus housing	.77	.57-1.04	2.85	.10	1.38	.82-2.32	1.45	.23	-.04	.04	-.03	.33
Alcohol use frequency	1.27	1.16-1.40	25.82	<.01	1.26	1.08-1.47	8.40	<.01	.09	.01	.21	<.01
Age of alcohol onset	.94	.90-.99	6.84	.01	.92	.86-.98	7.29	<.01	-.03	.01	-.12	<.01
Happy hour drinking	1.29	.97-1.70	3.07	.08	2.18	1.30-3.65	8.75	<.01	.14	.04	.13	<.01
Constant	.41				.02				1.70			
Model χ^2^	66.23				71.55				F	1.95		
Pseudo R^2^	.08				.13				R^2^	.10		

Gender emerged as one of the strongest predictors of self-reported driving under the influence with males being far more likely to report the behavior. Those who drank more frequently, initiated alcohol use earlier, and increased their drinking during happy hours were also significantly more likely to report driving under the influence. Other than gender and employment status, none of the other demographic factors had a significant association with driving under the influence while controlling for frequency of use, onset, and happy hour drinking.

Model 2 replaced self-reported driving under the influence with alcohol-related arrest/citation. Males, Whites, and older participants were more likely to report an arrest or citation. Similarly, those who drank more frequently and those who initiated alcohol use earlier were more likely to report an arrest controlling for other factors. Happy hour drinking changes were not associated with an altered likelihood of arrest or citation. This remained true in Model 3 when the dependent variable was limited to arrests related to operating a vehicle while impaired.^c^

Models 4 and 5, shown in Table 3, examined the negative outcomes of risky sexual behavior and fighting while drinking, respectively. The models depict that males were significantly more likely than females to engage in unprotected sexual intercourse with a stranger while drinking and fight while drinking while controlling for other factors. None of the other demographic factors had a significant association with either dependent variable at the .05 level. Those who reported drinking more frequently and those who reported initiating alcohol use at an earlier age were significantly more likely to engage in both of the behaviors. Altered happy hour drinking was significantly associated with a greater likelihood of fighting while intoxicated; however, for unprotected sexual intercourse with a stranger while drinking, altered happy hour drinking approached but did not reach significance (p = .08).

The final model investigated general alcohol-related problems using ordinary least squares regression. Though no demographic factors were associated with the alcohol-related problems scale, drinking frequency, age of onset, and happy hour drinking changes all had significant relationships with the measure while controlling for one another and the demographic variables.

## Discussion

Restrictions on happy hour and other bar specials are a direct form of alcohol price control, and these policies appear to be constantly changing across the United States. In order to inform policymakers and reduce negative consequences associated with drinking, it is essential to gain a better understanding of the impact drink specials have on alcohol consumption and on specific types of alcohol-related harms. Our study indicates that certain individuals do alter their drinking behavior in the presence of happy hour specials and that the altered behavior is related to specific negative consequences such as operating a vehicle while intoxicated and getting into physical altercations. These associations were substantively large. Odds ratios of 1.88 and 2.18 indicate that altered drinking behaviors during happy hour roughly doubles the odds of getting behind the wheel or into a fight while drunk. Changes in happy hour drinking, however, were not associated with an altered likelihood of alcohol-related arrest or citation. As would be expected, other factors such as gender and frequency of alcohol use were also related to the negative outcomes, including self-reported driving under the influence, fighting while drinking, the alcohol-related problems scale.

Among our sample, several demographic groups (i.e., women, individuals under 21, non-athletes, members of Greek-affiliated organizations, those with the lowest or no GPAs, more affluent students, unemployed, and students living on campus) were significantly more likely to alter their drinking behavior in the presence of happy hour specials, suggesting that the economic factors may have a differential impact on drinking behaviors. Most notably, those students at increased risk for negative outcomes and victimization, namely women and novice drinkers, were particularly affected by happy hour specials. The odds of women reporting altered drinking during happy hour were 1.45 times that of male respondents. For women, an indirect relationship with the happy hour may be occurring as they may be receiving and not necessarily buying their own drinks during the discount period. Conversely, the special itself could be for females only (e.g., ladies night, ladies happy hour). Those who were not yet 21 years of age were far more likely to increase their drinking than the age groups able to legally drink. Additionally, although respondents with the lowest GPAs reported increases in drinking during happy hour the most^d^, those with no GPA were the second highest to report increases their drinking behavior during happy hour, and the lack of GPA may be related to age. Those without a GPA most likely consist of true freshmen (not transfer students); these freshmen are typically younger than their more senior classmates. Further, the odds of underclassmen changing their drinking during happy hours were three times that of upperclassmen. These results may be problematic since this age group is more likely to be less experienced with alcohol consumption and less able to moderate drinking.

Based on the results, responsibility and a counterintuitive financial reason may be related to changes in happy hour drinking behavior. Non-athletes, single individuals, and those who were unemployed or employed part time were significantly more likely to increase their drinking behavior during happy hour specials. With happy hours beginning in the late afternoon, opportunities for participation may be limited for those with families, full-time work duties, or athletic responsibilities. They may be altering their behavior more when attending happy hours, but attending happy hours more rarely than other individuals. Although lower prices during the specials may drive some of the increased drinking behavior, individuals with higher family incomes were significantly more likely to increase their drinking behavior in the presence of happy hour specials. This finding may have emerged because those from more affluent families may have more disposable income than students with less affluent families. Those with less disposable income may less inclined to go out and drink at the bars because drinking at these establishments, even in the presence of happy hour specials, is more expensive than drinking at home in the United States.

To determine whether the relationship between happy hour utilization and negative consequences was contingent upon whether individuals were of legal drinking age, we examined additional models split by age. Wright’s comparison of coefficient tests [35] indicated that the relationship was not moderated by legal drinking age with one exception. Drinking changes due to happy hour specials were more strongly and significantly associated with unprotected sex with a stranger for those over 21 years of age (Z = 2.395, p = .017). For this population, the happy hour coefficient did reach significance (b = .504, OR = 1.656 (CI 1.084-2.258), p = .020) whereas it did not among younger students (b = .013, OR = 1.014 (CI.687-1.495), p = .946). However, we recommend cautious interpretation of this finding since our analysis could not control for relationship status, which may be correlated with age.

Although not all risk factors can be addressed by policy, some factors can be if they are identified, and this study did identify several modifiable risk factors. For example, while gender may have the strongest association with driving under the influence, risky sexual behavior, and fighting, it is not a modifiable factor. However, our findings indicate that some modifiable factors were also associated with these negative consequences and the alcohol-related problems scale. Specifically, happy hour utilization and the frequency of alcohol use are modifiable factors that were associated with fighting while drinking, driving under the influence, and the alcohol-related problems scale. The opportunities to engage in happy hour drinking and frequent alcohol consumption are perhaps the most accessible modifiable factors.

The factors related to legal consequences were different than those previously discussed for non-legal consequences. The modifiable factors previously mentioned were not significantly related to DUI citations or arrests, but age of onset and frequency of use were related to alcohol-related arrest. Changes in happy hour drinking were not even significantly related to alcohol-related arrests or citations independent of demographic and other controls. Happy hour specials appear to only be related to increases in non-legal consequences; this should be viewed as worrisome given that those that alter their drinking behaviors during happy hours reported greater involvement in two illegal activities (i.e., impaired driving and physical fights).

### Limitations & future research

Because the data utilized in the present study are restricted to college students in one community, we cannot generalize our results to the behaviors of young adults elsewhere. Our data suggest that underage drinking at bars, whether with false identification or allowed by relaxed age-verification practices, is almost normative at the study location. More than half of the sample over the age of 21 reported having consumed alcohol before they were 21. Among the underage portion of the sample, nearly half are current alcohol users. Results may not be so robust in areas with stricter enforcement of underage drinking regulations. Similarly, college students are likely to be distinct from other young adult populations in a variety of ways, so our data cannot be used to reach conclusions about adults more generally. However, as previously mentioned, many alcohol studies focus on college populations [2-5,8,9,12,14,16,36-39] because nearly half of college students participated in binge drinking in the last year [11-14] and approximately 600,000 traumas and 1,700 deaths were alcohol related in the college population each year [2].

The cross-sectional nature of the data is a limitation. It is possible that the measured outcomes preceded happy-hour-affected alcohol consumption. Perhaps individuals with risky drinking indicators (e.g., age of onset) who have already experienced negative consequences could be encouraged to continue with an existing pattern of hazardous drinking behavior by the presence of happy hours. However, we feel that it is more reasonable to expect that the way in which a person consumes alcohol affects whether the individual engages in fighting, unprotected sex, and driving under the influence.

We additionally note limitations with the self-report measures. Specifically, the self-reported DUI measure was created using survey items that did not directly define a standardized drink or length of a drinking episode; it only asked whether one drove after consuming five or more drinks. More generally with regard to self-report measures, there is concern with recall bias, social desirability, and self-identity protection issues. Further, concern with the method being able to elicit truthful responses on sensitive items (e.g., illegal behavior) has been expressed. However, the survey that produced this data did not ask for names, student identification numbers, or contact information. Further, research has either found no strong effect in the way questions were administered (anonymous, non-anonymous, questionnaires, interviews) or slight benefits with the self-administered questionnaires (see [40], for a discussion of the development and impact of self-report measures). Yet, recent research does indicate that recall of intoxication levels further away from the time of consumption may not be accurate [41]. Further, a weakness of many alcohol studies, including this one, is “the lack of event-specific analyses to directly link bar-sponsored drink specials with patron alcohol use” ([42]:206). Thus, it should be noted as a limitation that the self-report measures in this study did not utilize calendars, diaries, or specific and recent recall periods.

We have several recommendations for future research. First, this study did not assess the impact of happy hour advertisements, which are the focus of many states’ regulations as opposed to the specials themselves. Second, drinking games (e.g., beer pong tournaments, trivia, shot challenges) and celebratory events such as football games have been shown to produce higher rates of consumption (e.g., [36-38,43,44]), which are often tied to happy hour specials in college towns*.* Future research should explore the effects of advertising, specific happy hour times, and other types of alcohol specials (e.g., game-based, event-based, and all-you-can drink specials) on drinking behaviors and negative consequences. Additionally, research should address the causality of the relationships discovered in our study, such as by collecting data on students both before and after specific drinking events. The findings from our study justify future research into the nature of these relationships. While our results may suggest that regulating and restricting happy hour drink specials may reduce a number of alcohol-related problem behaviors, we challenge future research to further evaluate this relationship and explore ways in which communities can lessen these effects.

## Conclusion

This study indicates that happy hour specials may influence the behavior of certain groups more than others, and some findings from this study are in accord with previous research findings. Our study and previous research (e.g., [18]) support the claim that more individuals increase their consumption than not in the presence of happy hour specials. Further, our results regarding age are similar to findings that indicate the alcohol price may affect the young adult population [8,9] and adolescents [10] more so than other age groups. By developing a better understanding of whom is affected by drink specials, it may be possible to focus precautionary and educational campaigns on those most likely to modify their behaviors in the presence of these specials. For example, young adults drinking underage, women, and those living in college dormitories may benefit from targeted social marketing campaigns [45,46]. Social influence media campaigns have shown to significantly reduce binge drinking and frequency of consumption in college populations [45].

This study, as well as other research (e.g., [47-53]), have found that age of onset is related to increased consumption and negative consequences. Thus, stricter enforcement of underage drinking laws might provide a way to reduce alcohol-related problems, especially among young drinkers. However, research on stricter enforcement is mixed, as the actual level of enforcement may not be representative of the perceptions of enforcement [54]. Research indicates that increasing sanctions and monitoring of establishments (rather than targeting individuals) may prove beneficial [55]. Additionally, while establishments employ happy hours to increase patronage during off-peak drinking hours, research on bar density has found a positive relationship between adolescents’ perceptions of alcohol availability and parental approval of consumption [54]. In light of this finding, an additional option may be to allow happy hours and drink specials at establishments where only those at least 21 years of age are permitted to enter.

Additional group-related results incite policy discussion. Individuals without athletic or full-time employment commitments were significantly more likely to increase their drinking behavior during happy hour specials, which may be related to the opportunity to attend happy hours specials as previously discussed. Offering alternative recreational activities such as discounted movie showings and dinners or athletic and club events without alcohol during the same time as happy hour specials in the area may prove effective and garner community support.

This study in conjunction with other research suggest that policymakers may affect certain negative consequences by altering local policies related to alcohol sales and opportunity to engage in alcohol use. Our findings lend some support to the idea that happy hours and other bar specials should be carefully regulated in an effort to reduce high consumption and the related negative consequences. In addition to the policies previously mentioned, encouraging responsible beverage service and employing harm reduction approaches such as offering or increasing public and free transportation, security, designated driver/responsible drinking companion programs, free water and bar snacks, advice on consumption, and warnings/unit information on containers [56-59] may also be more effective policy strategies and have more public support.

## Endnotes

^a^The majority of missing cases resulted from absences from class. Students were instructed to simply return the blank survey if they were unwilling to participate. Less than 50 students chose this option. We were not allowed to collect any data about these individuals. The response rate estimate represents the number of completed surveys divided by the total number of students enrolled in the courses at the end of the enrollment period adjusted for students enrolled in multiple selected courses. The 80.4% response rate is viewed as a conservative estimate since a more accurate denominator would be the number of students enrolled in the courses on the day of administration. We do not have access to lists of students removed from courses due to non-payment, medical or military leave, mid-semester expulsion, or course or university withdrawal.

^b^The four age groupings were created to represent non-legal drinkers (17–20), those new to the legal drinking (21 and 22), older traditional students (23–25), and non-traditional students (older than 25).

^c^Model 3 employed King and Zeng’s [60] correction for rare events because, unlike other dependent variables, DUI citations were reported by less than 5% of the sample.

^d^All five individuals who reported a GPA in the lowest category (0.0-1.0) indicated an increase in drinking behavior during happy hour.

## Competing interests

The authors declare that they have no competing interests.

## Authors’ contributions

JS and BM gathered the data. JB and JS originated the premise of the paper. JS carried out the analysis and drafted the methods and results, which were revised by JB. JB drafted the rest of the manuscript with BM contributing to the discussion and conclusion. All authors participated in proofreading the manuscript, and JB conducted the major and final edits, as well as formatting, of the manuscript. All authors read and approved the final manuscript.
